# The impact of criminal law regulation-based business environment optimization on entrepreneurial spirit and enterprise development

**DOI:** 10.3389/fpsyg.2022.944146

**Published:** 2022-07-29

**Authors:** Xianzhen Liu, Shuai Li

**Affiliations:** Law School, East China Normal University, Shanghai, China

**Keywords:** Criminal Law Regulation, business environment, entrepreneurial spirit, enterprise development, structural equation model

## Abstract

The purpose is to explore the impact of the business environment optimization by Criminal Law Regulation (CLR) on Entrepreneurial Spirit (ES) and Enterprise Development (ED) and to provide a reference for subsequent related research. Based on this, this work first makes a detailed analysis of the business environment and CLR. Second, the research hypotheses are put forward, and the conceptual model is proposed. At the same time, a Questionnaire Survey (QS) is designed to analyze the business environment, ES, and ED, and their relationships. Finally, a Structural Equation Model (SEM) is constructed and the CLR-optimized business environment is used as the intermediary variable to explore the impact of the business environment on ES and ED. Then, 200 QSs (recovering 192 valid ones) are distributed to investigate entrepreneurs' attitudes toward ES and ED in different regions. Statistical analysis and independent *t*-tests are performed on the survey results to judge the relationship between variables. The results of empirical analysis show that (1) The significance coefficient P of ES and ED is 0.005 < 0.01, and the scores of ES and ED of large enterprises are 132.7864 and 142.3243, respectively, which are the highest. Therefore, CLR-optimized business regulation has a significant positive impact on the ED. (2) The influence coefficient of CLR-optimized business regulation and ES is 0.60, and the influence coefficient of CLR-optimized business regulation and ED is 0.75. Therefore, CLR-optimized business regulation plays a positive role in the development of ES. CLR-optimized business regulation plays a regulating role between ES and ED. (3) CLR-optimized business regulation has a significant positive impact on the formation of ES. The policy enlightenment of this work mainly has three points. First, optimizing the business environment can stimulate and protect ES, thus improving the quality of economic growth. All regions should promote “mass entrepreneurship and innovation” and high-quality economic development by improving the convergence of economic policies and building a legal and market-oriented business environment. Second, all regions should implement dynamic and differentiated policies to optimize the business environment's spatial pattern in Chinese cities. Third, there is a need to further strengthen the construction of new infrastructure through cutting-edge information technologies, such as Fifth Generation (5G) mobile communication, Big Data, and Artificial Intelligence (AI).

## Introduction

Since the publication of the Business Environment Report in 2003, the business environment has begun to catch the vision of the public and researchers (Indrajit et al., 2021). As the market reform deepens, Market Economy has gradually become the dominant economic form, where the business environment plays a key role. Accordingly, scholars extensively research issues related to the business environment (Abu-Rumman, 2021). In particular, with the ongoing global Corona Virus 2019 (COVID-19) pandemic, the global economy is experiencing a recession. As a result, China's economy has entered a dual economic cycle strategy: the internal economic cycle as the main body and supplemented by the global economic cycle. Thus, only a good business environment can promise sustainable economic development (Yustian, 2021). The regulations on optimizing the business environment promulgated by the State Council define the business environment as “the institutional factors and conditions involved in the market economic activities of enterprises and other market subjects,” including the protection of market subjects, economic environment, administrative guarantee, market supervision, and legal protection (Rangel-Preciado et al., 2021). The business environment refers to various institutional arrangements that affect the activities of market subjects, including institutional mechanisms, laws and regulations, and rules and procedures (Sobehart, 2021). Here are some main features of the business environment. The business environment is an institutional environment that enterprises must abide by in their daily business activities. It has an essential impact on the daily development of enterprises. As a part of the market, the business environment must maintain its unique norms, justice, and stability. The impact of the business environment runs through enterprises' whole process, from registration to bankruptcy. All behaviors of enterprises in the market should follow the business environment. The economic, temporal, and opportunistic costs paid by the market to create a business environment are called institutional transaction costs (Nohoua, 2021).

However, China's structural problems are becoming much more serious, and entity enterprises are gradually entering the financial and real estate industries. The lack of Entrepreneurial Spirit (ES) might be the main reason for the slow development of entity enterprises (Ibrahim et al., 2021). Foreign studies have shown that ES is an important driving force in promoting economic development (Gu et al., 2021). Hofland-Mol (2022) believes that creative industries need entrepreneurship to survive and develop in the global economy. Chakraborty et al. (2022) believed that ES could mediate and regulate through the consumption value theory to promote agricultural product consumption. Therefore, it is of practical significance to study and optimize the business environment for ES and Enterprise Development (ED) (Nam and Bao Tram, 2021). For a long time, in the ES and ED research, most economists have believed that ES cultivation is an important guarantee for economic development. They analyze and demonstrate their views through the empirical test. Some scholars claim that ES affects the coordinated development of real and virtual economies through entrepreneurs' behavioral decisions. The lack of ES is an important reason for uncoordinated economic development (Iqbal et al., 2021). The above literature initially explores the impact of ES on ES but ignores the impact of the business environment on ED. Most literature only concentrates on the mechanism of financial environment or financial, ecological environment on ES and ED. The legalized business environment is the booster of China's high-quality economic development. In optimizing the business environment, legal norms can reduce policy uncertainty and the randomness of reform, enhancing institutional constraints and stabilizing market expectations (Chen et al., 2021). Therefore, the legalization of the business environment is inseparable from a set of effective, fair, and transparent laws and regulations. Chinese central and local governments have issued relevant laws and regulations to optimize the business environment. Most of these laws and regulations have made provisions for constructing the Rule of Law (RoL) in the business environment to create a fair and orderly market environment. They provide efficient and convenient government services and strengthen legal and standardized supervision and law enforcement. Furthermore, these laws provide strong support and guarantee for optimizing the business environment at the institutional level (Nahaei and Bahrami, 2021). Therefore, optimizing the business environment through Criminal Law Regulation (CLR) provides a legal guarantee for ES and ED.

It has become the action consensus of the government and all sectors of society to promote high-quality economic development by planting fertile soil for the growth of entrepreneurs and stimulating and protecting ES to promote sustainable economic growth. To this end, the Communist Party of China (CPC) Central Committee and the State Council have successively issued a series of regulations and important documents. They include the Opinions on Creating a Healthy Growth Environment for Entrepreneurs, Promoting Excellent ES, and Giving Better Play to the Role of Entrepreneurs issued in 2017. These documents affirmed the role of entrepreneurs for the first time. At the end of 2019, the Opinions on Creating a Healthy Growth Environment for Entrepreneurs were launched to support private enterprises' reform and development. It further clarified that private enterprises could fully unleash their creative vitality by creating a market-oriented, legal and international business environment. The Regulations on Optimizing the Business Environment were implemented in January 2020 and filled the gap in domestic legislation in the field of the business environment. At the same time, China has made significant progress in optimizing the business environment and stimulating market vitality. Obviously, improving the business environment and the ES growth has become a typical fact that China's economy has entered a high-quality development stage.

In the context of economic slowdown and rising unemployment in China, stimulating ES, activating market competition, and driving employment is important to economic growth (Tien et al., 2021). In view of the critical role of CLR-based business environment optimization in ES, it is necessary to investigate the ES and ED in China under different business environments. The research finding can provide reference opinions for improving China's business environment and promoting enterprise economic growth.

## Conceptual foundation and model building

**Figure 1:**
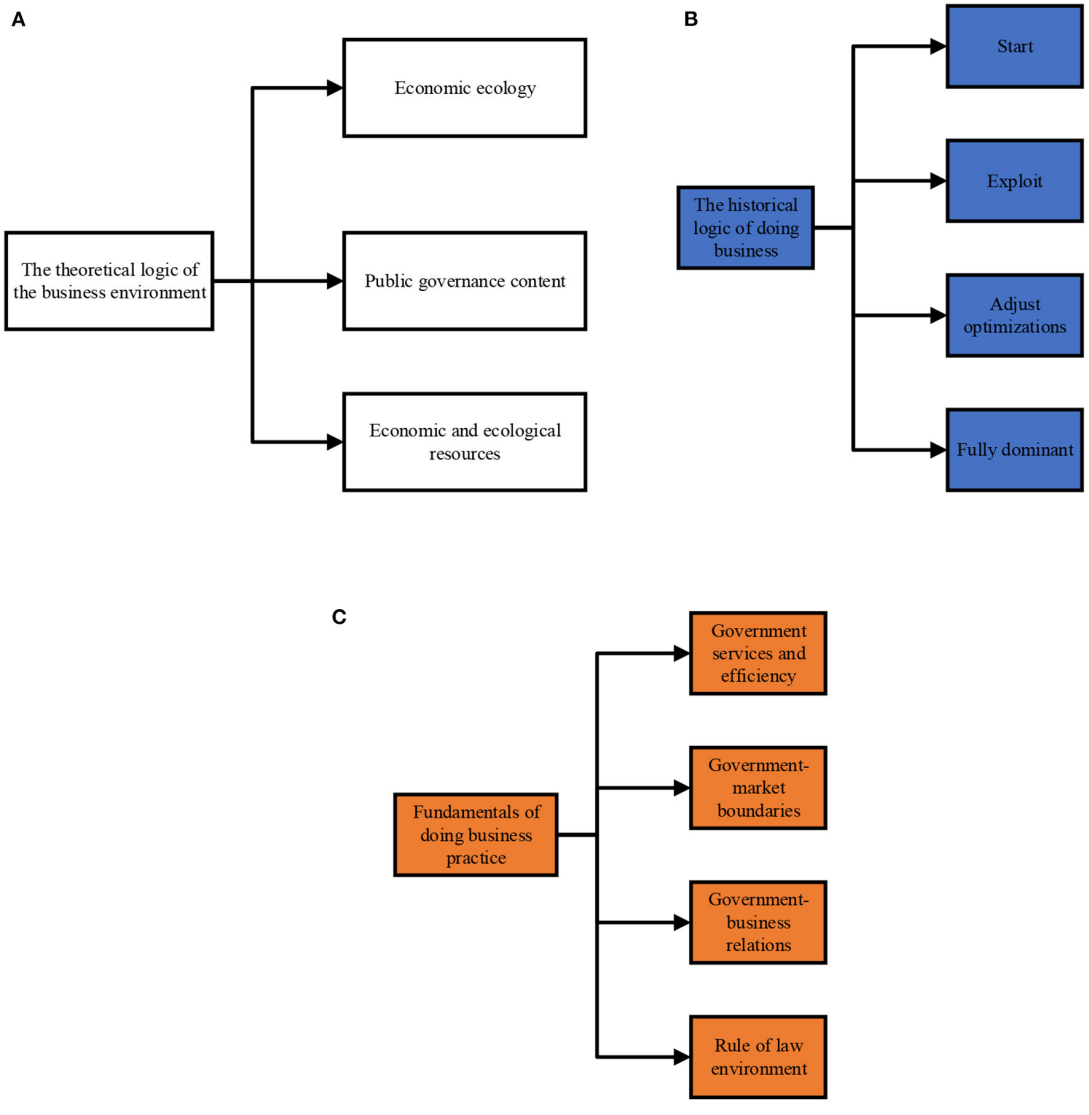
Business environment content **(A)** Logical content of business environment theory; **(B)** Logical content of business environment history; **(C)** Logical content of business environment practice.

Business environment refers to the collection of relevant external factors and conditions, such as government environment, market environment, legal environment, and humanistic environment, involved by market subjects in the process of access, production, operation, and exit (Huang et al., 2022). It is a systematic project involving many economic and social reform fields and opening to the outside world. The quality of a region's business environment directly affects the number of investment attractions. The operating enterprises in the region ultimately have an important impact on economic development, fiscal and tax revenue, and social employment (Guenther and Guenther, 2022). Generally speaking, legal, political, economic, and social elements affect enterprise activities. A good business environment embodies soft economic power. It is an important aspect of improving the comprehensive competitiveness of a country or region (Bratianu et al., 2021). The nature of the business environment is depicted in Figure 1.

As shown in Figure 1, from the perspective of theoretical logic, the business environment is essentially an economic ecology, an important part of government public governance, and non-exclusive and non-competitive economic and ecological resources shared by all market entities. The business environment determines that the government, which manages and serves the society, has the leadership responsibility for the construction and overall planning of the business environment (Leipold, 2021). From the perspective of historical logic, the role of the Chinese government in the construction of a business environment has experienced four stages: starting, pioneering, adjustment and optimization, and overall leadership (Badi et al., 2021); From the perspective of practical logic, the optimization of China's business environment mainly includes government performance services, government efficiency, the boundary between the government and the market, a close and harmonious new government enterprise relationship, and the legal environment (Lin, 2021). The domestic research on the business environment is vibrant, mainly reflected in four dimensions. One is the discussion on the value and significance of the business environment. The second is the research on the quality evaluation of the business environment. Thirdly, it discusses the elements of the business environment, and fourthly, it is the proposed strategy to optimize the business environment (Angelino et al., 2021). Cultural capital, government behavior, and financing channels are the main path for the business environment to affect ES. The essence of ES is the accumulation of cultural capital centered on the expansion and innovation of values. The diversified and inclusive urban cultural atmosphere is significant in cultivating ES. In terms of government behavior, excessive administrative regulation will increase the cost that enterprises expenditure to maintain the relationship between government departments and address related affairs. The lack of a property rights system will cause entrepreneurs to have negative expectations of the “plunder” they may bear in the process of entrepreneurship. These factors will negatively affect enterprises' confidence, thus inhibiting the ED and the development of innovation activities. The imperfection of financial services will reduce the possibility of enterprise financing and increase the financing cost, which will also be detrimental to the growth of entrepreneurship. It can be found that the improvement of the soft business environment will help enterprises reduce transaction costs, thus enhancing the urban ES.

China's business environment planning aims to establish a more legalized, international, and liberalized business environment. Specifically, legalization (Rule of Law) in a business environment refers to a series of specific laws, regulations, and regulatory procedures that are effective, fair, just, and transparent (Gill and Ramachandran, 2021). Internationalization is to establish a Market Economy operation mechanism and system consistent with international practice and World Trade Organization (WTO). For example, the business environment of Small and Medium-sized Enterprises (SMEs) should consider the market environment, policy and government environment, social service environment, financing environment, and RoL environment to promote investment liberalization (Xie and Zhang, 2021). Investment liberalization is a higher requirement for the development of economic globalization, which has three requirements for the business environment. First, there is a need to change the concept of market management, namely, changing from product supervision to enterprise supervision. Second, there is a need to change the trade structure: liberalizing the existing trade barriers and quota restrictions on trade and investment. Third, policy direction must also be changed. The relaxation of foreign exchange and tax policies will enable more open and internationally competitive enterprises (Kandilov et al., 2021).

**Figure 2:**
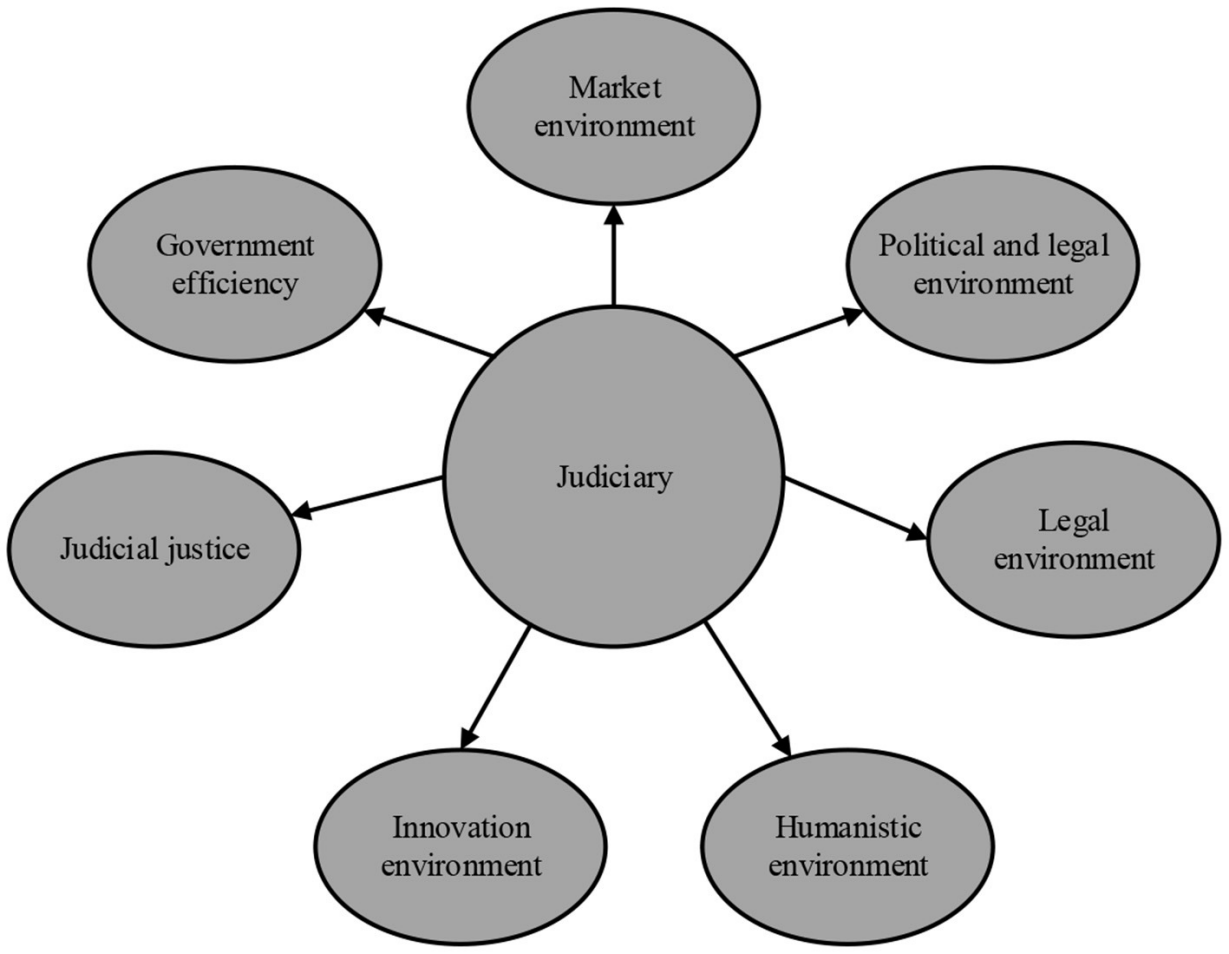
The CLR-based business environment optimization model.

To date, the CLR-based business environment optimization is mostly carried out from three aspects: optimizing the market environment, optimizing the government environment, and optimizing the legal environment. Of these, the core of optimizing the market environment is to ensure the fairness and order of the market environment. The core of optimizing the political environment is to provide efficient government services. Lastly, the core of optimizing the legal environment is strengthening fair and standardized supervision and law enforcement (Oncioiu et al., 2021). The CLR-based business optimization model is illustrated in Figure 2.

In Figure 2, in the CLR-based business environment optimization model in China, the judicial department plays a major guiding role and can have a certain impact on other environmental elements, such as market, RoL, policy, innovation, and humanities, and can be decisive (Yan and Qi, 2021).

## Research methodology

### Research model and hypothesis

The possible marginal contributions of this work are as follows. First, from the perspective of research, it enriches the relevant research on how the business environment affects the quality of economic growth from the dimension of ES growth. Second, in terms of the evaluation system, this work attempts to build a business environment index for cities at the prefecture level and above and analyze its temporal and spatial characteristics. Compared with previous studies on large and medium-sized cities or inter-provincial levels, it can observe the evolution of China's business environment from a longer-term and a subtler spatial scale. Third, in terms of empirical methods, on the one hand, higher quality industrial and commercial registration data and innovation capability index are used to measure urban ES. Compared with the traditional statistics of employees and the number of patents, the information is more comprehensive, and the measurement deviation is smaller. On the other hand, this work starts from the quasi-natural experiment of commercial system reform. Then, the Questionnaire Survey (QS) method is used to explore how business environment optimization affects the growth of ES. Furthermore, the systematic empirical research on promoting the quality of economic growth provides a robust estimate. Also, it provides a high-quality development perspective of policy effect assessment for the measures to optimize the business environment in recent years.

The relationship between the variables studied is as follows:

(1) CLR-based business environment optimization and ED

CLR-based business environment optimization is an important factor affecting economic development. A good business environment regulated by criminal law will help promote the steady development of the market. The internal mechanism of CLR-based business environment optimization to affect the ED is explained below. CLR-based business environment optimization helps reduce the operating costs of entity enterprises, speeds up the allocation efficiency of productive capital, and ensures the fairness of the market economy and the stability of the financial market. A good business environment is conducive to industrial up-gradation. CLR-based business environment optimization can help standardize the market economy competition, which will force enterprises to improve their competitiveness.

Based on this, the following hypotheses are proposed:

H1: CLR-based business environment optimization can significantly promote the ED.

(2) ES and ED

Extensive studies have shown that ES is an important driving force for economic development. The relationship between ES and economic growth has long attracted scholars' attention. However, the literature on Total Factor Productivity (TFP) started to grow in the early twenty-first century. The average TFP of start-ups in the UK manufacturing industry is significantly higher than that of incumbents and quitters, which infers a positive correlation between ES and TFP. In addition to the evidence at the enterprise level, empirical research based on the German background also finds that ES can improve regional TFP. These scholars believe that the knowledge spillover effect is the main mechanism. The empirical test from the industrial level proves that ES significantly improves the TFP of Chinese industry through the knowledge spillover effect. The above research mainly considers the role of ES in promoting TFP by driving technological progress and focuses more on Schumpeterian ES characterized by “creative destruction.” As the micro main body of the market economy, entrepreneurs have an important influence in searching for business opportunities, ensuring market stability, and promoting industrial upgrading. For ED, it is imperative to develop potential markets further, expand the market scale, and promote product upgrading to meet consumers' demand for high-quality products. The development of potential markets, the expansion of the existing market scale, and the emergence of new products and services will all lead to an increase in the number of producers and the application of new technologies. The promotion of ES will promote the emergence of new enterprises, new technologies, and new products and promote ED.

Based on this, the following hypotheses are proposed:

H2: ES has a certain degree of positive impact on the ED.

H2a: ES has a significant positive impact on ED.

H2b: Innovation spirit has a significant positive impact on ED.

H2c: The adventurous spirit has a significant positive impact on ED.

H2d: The tolerance spirit has a significant positive impact on ED.

(3) CLR-based business environment optimization and ES

CLR-based business environment optimization has an important impact on the formation of ES. The business environment is an important way for ES to affect the development of the real economy. Meanwhile, RL-based business environment optimization can create a good market environment for entrepreneurs' financing services, market exploration, and product development. It provides more entrepreneurial opportunities and stimulates entrepreneurial enthusiasm. At the same time, ES can affect ED through the business environment.

Based on this, the following hypotheses are proposed:

H3: CLR-based business environment optimization has a significant positive impact on ES.

**Figure 3:**
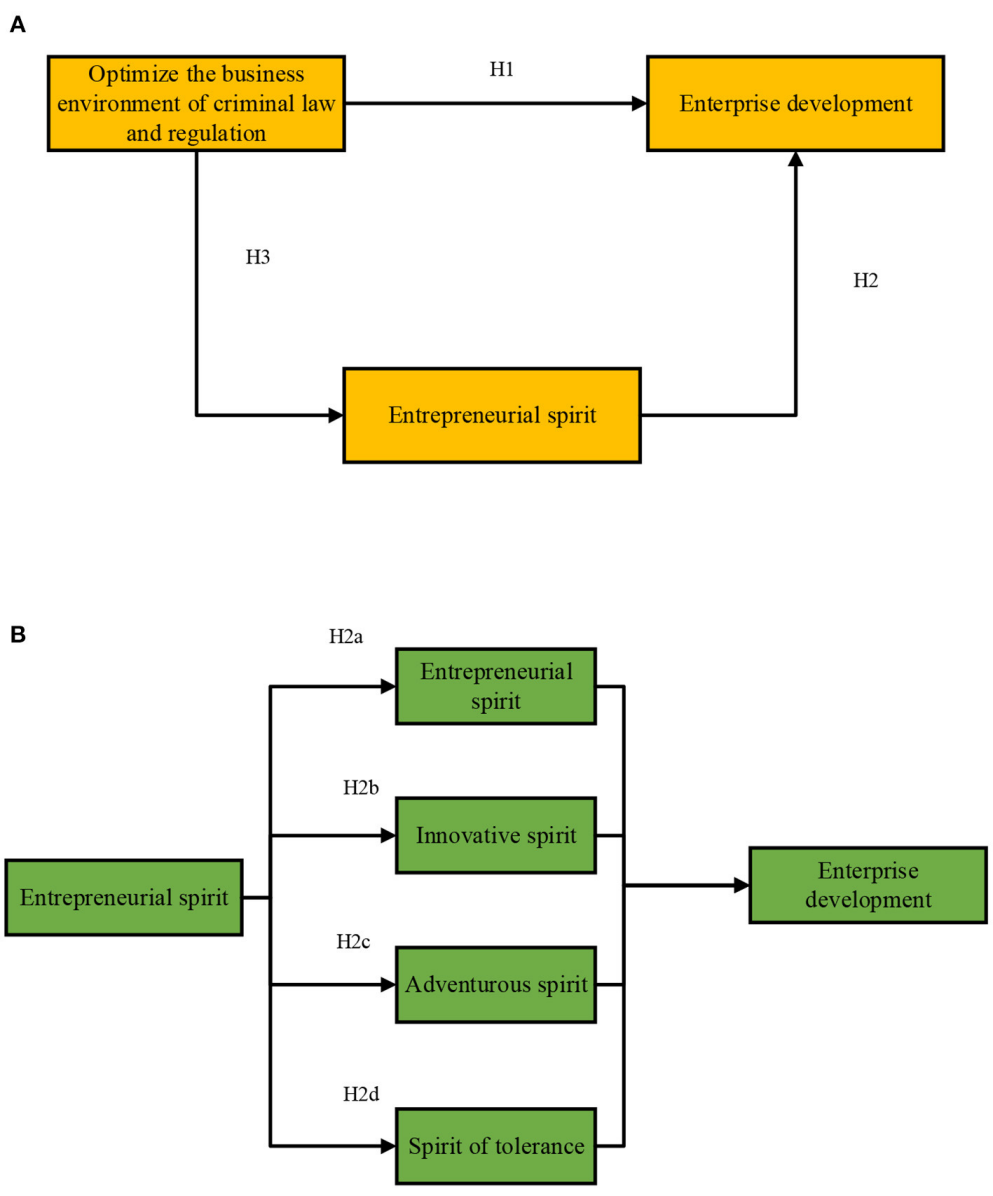
The conceptual model of CLR-based business environment optimization, ES, and ED. **(A)** The overall conceptual model diagram; **(B)** the conceptual model of ES and ED.

A conceptual research model is proposed based on the above research hypotheses, as shown in Figure 3.

### Questionnaire survey (QS) design

**Figure 4:**
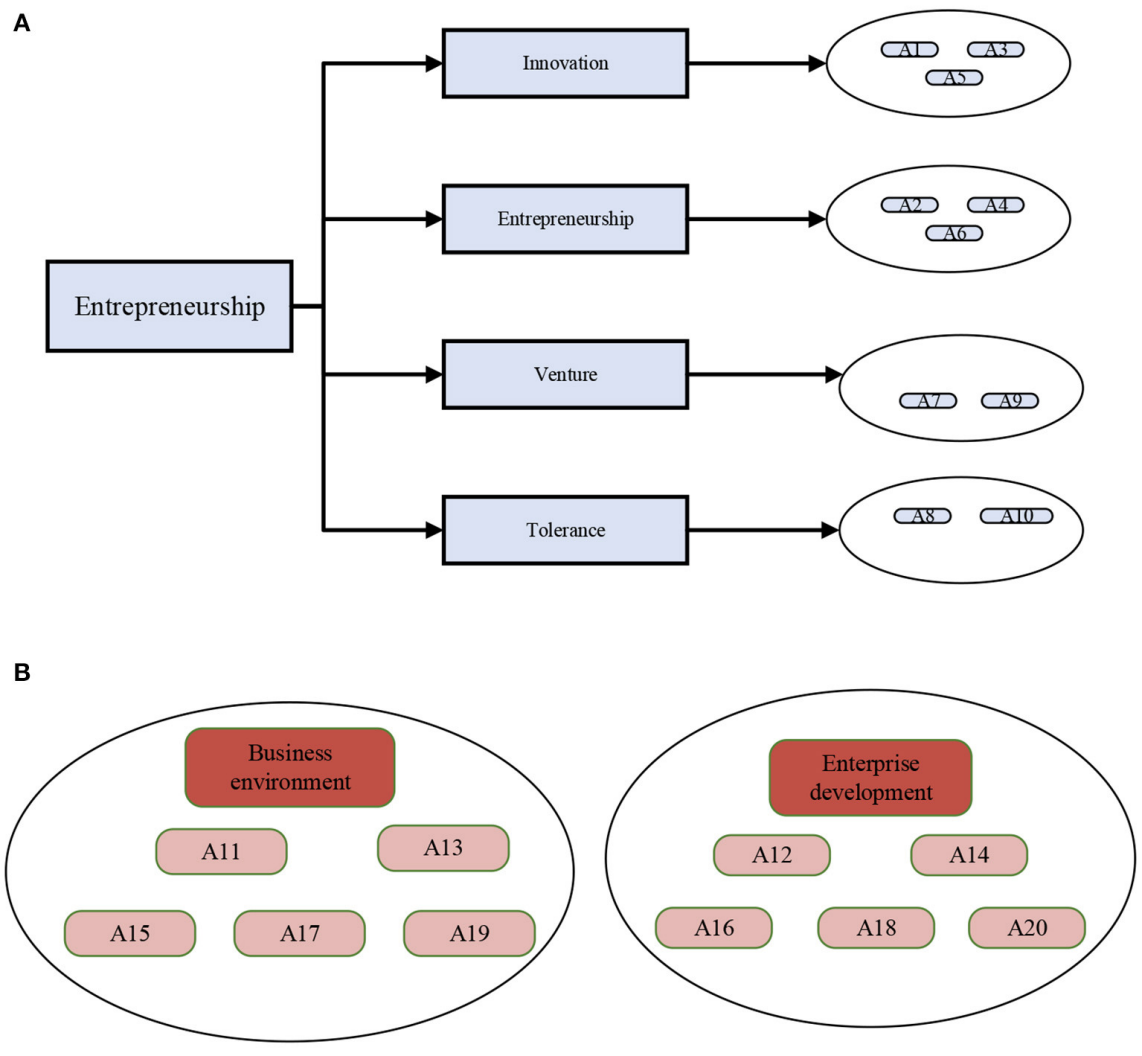
Distribution map of items in the research scale **(A)** Distribution map of ES Scale; **(B)** Distribution of CLR-based business environment optimization Scale and ED Scale.

For the research on the correlation between ES, ED, and CLR-based business environment optimization, this work modifies the QS scale by referring to the existing mature Scale and setting up 20 items. Specifically, the ES Scale includes four dimensions: ES, innovation, adventure, and tolerance, with 10 items. The ED Scale covers five items. CLR-based business environment optimization Scale involves five items. Then, Likert's 5-point Scale scores the questionnaire. The lowest score is 1, meaning very inconsistent, and the highest score is 5, meaning very consistent. The research scale is laid out in Figure 4:

The original input of the model is QS data. The valid QS data are sorted, coded, and entered into Statistical Product and Service Solutions (SPSS) 26.0 and archived. Each option is coded as 1-5 in turn in the data processing. SPSS 26.0 and Analysis of Moment Structure (AMOS) 19.0 are used for data analysis. Specifically, AMOS analyzes the structural validity of the Scale. It is fully functional and can help users easily create various models, facilitate AMOS analysis, and provide model analysis and statistics functions. These functions can help users automatically analyze models. Meanwhile, it can meet users' requirements to calculate new fitting measures. After inputting the QS data into AMOS software, the simulation fitting index is obtained. The designed data analysis methods include reliability and validity analysis, basic descriptive statistics, independent sample *t*-test, one-way Analysis of Variance (ANOVA), and path test.

### Data acquisition and statistical analysis

**Figure 5:**
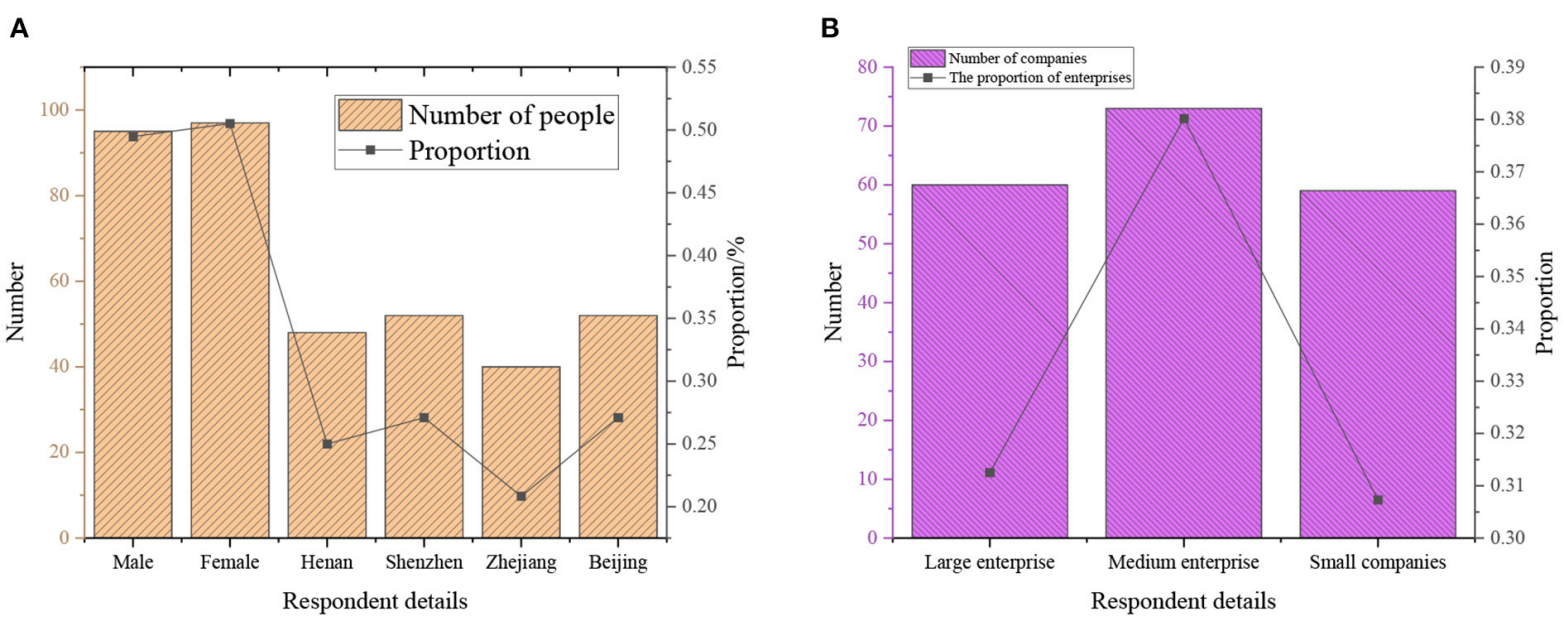
Detailed distribution map of respondents. **(A)** The number and proportion of gender and city; **(B)** The number and proportion of business types.

Here, online QSs are distributed to collect data to verify the establishment conditions of the theoretical model more comprehensively. First, a pilot is conducted in the city. Then, 50 QSs are distributed, collecting 46 valid ones. The QS is efficient, so the method is feasible. Entrepreneurs running different-sized enterprises in four different provinces and cities in China are selected as the survey objects. The survey method chooses random sampling. Overall, 200 QSs are released through Internet programs, and the background data were collated and analyzed to ensure the authenticity and reliability of the data. The QS is distributed for one week, and 192 qualified QS are finally collected backstage, with a qualified recovery rate of 96%. The online Questionnaire Star program is used together with a small-scale telephone interview for entrepreneurs who are not convenient to conduct the QS. The demographic variables of the research object are detailed in Figure 5:

## Experimental design and results

### Reliability and validity test of the scale

(1) Reliability analysis

Reliability analysis analyzes the consistency of the Scale. The Scale's reliability refers to the reliability, stability, and consistency of the survey results, namely, the accuracy (Teixeira et al., 2021). It is generally believed that reliability reflects measurement error or observation error: data change caused by a random error (Hou et al., 2021). Internal consistency reliability is usually expressed by the Cronbach'α coefficient (∈ [0,1]). The greater the Cronbach'α coefficient is, the higher its reliability is (Jasim et al., 2022). Generally, a Cronbach' α above 0.7 is needed for further data analysis. The specific calculation of Cronbach's α coefficient reads:


(1) $$\begin{array}{l} \alpha =\text{K}\left(1-\frac{\sum \sigma_{{\text{i}}^{2}}}{\sum \sigma_{{\text{T}}^{2}}}\right)/\left(\text{K}-1\right) \end{array}$$

Here, *K* is the number of questions on the Scale. $\sigma_{{\text{i}}^{2}}$ represents the variance of the score in question *i*. $\sum \sigma_{{\text{i}}^{2}}$ denotes the sum of variances of *K* items. $\sum \sigma_{{\text{T}}^{2}}$ indicates the variance of the sum of scores of all items.

(2) Validity analysis

Validity mainly evaluates the Scale's accuracy, validity, and correctness, namely, the error between the measurement results of the Scale and the real value of the target (Fan et al., 2022). Validity can measure the consistency between the actual measurement and the expected result (Tiger and Effertz, 2021). Validity analysis is usually simulated by a structural equation, in which the regression calculation of the simulation matrix reads:


(2) $$\text{F}(\text{S};\hat{\Sigma }=\text{tr}(\text{S}\overset{-1}{\Sigma })+\text{lg}|\hat{\Sigma }|-\;\text{p}$$

The calculation of Chi-Square Value (CSV) reads:


(3) $$\chi^{2}=\left(\text{n}-1\right)\text{F}(\text{S};\hat{\sum )}$$

In (3), **S** represents the actual data matrix. $\hat{\Sigma }$ is the hypothetical model matrix.

(3) Reliability analysis of internal consistency of Scale

**Figure 6:**
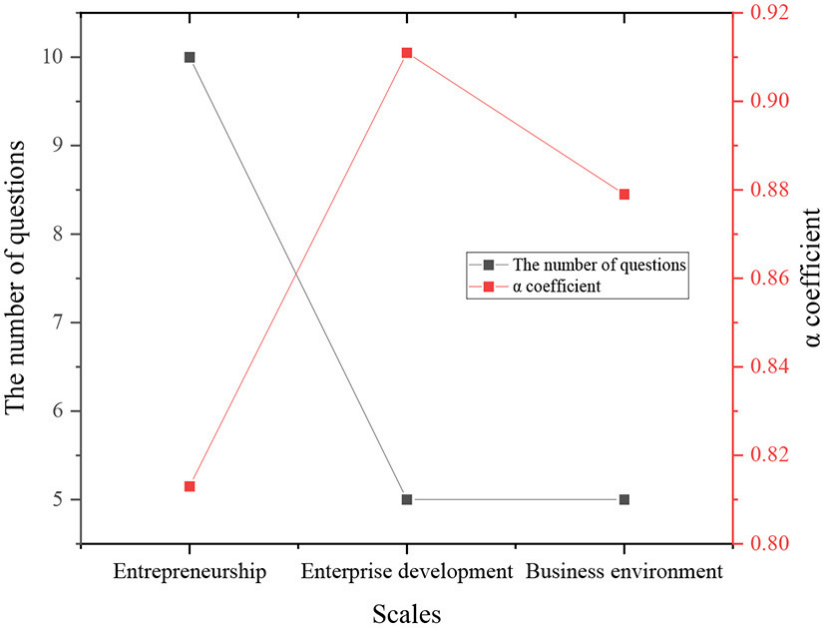
Internal consistency reliability of the Scale's α coefficient result.

The mean of CLR-based business environment optimization, ES, and ED scales' Cronbach'α coefficient for internal consistency reliability is presented in Figure 6.

According to Figure 6, the internal consistency coefficients of CLR-based business environment optimization Scale, ES Scale, and ED Scale are 0.813, 0.911, and 0.879, respectively, greater than 0.8. Thus, the three scales have good stability, and internal consistency can be used in the subsequent data analysis.

(4) Construct validity analysis of the Scale

After the QS results are inputted into AMOS, the simulation fitting index is obtained. The results are enumerated in Table 1:

**Table 1:** Model fitting index.

**Factor**	**RMR**	**GFI**	**AGFI**	**CFI**	**CSV**	**CSV/DF**	**RMSEA**
Acceptable cutoff	0.08	0.90	0.90	0.90	/	1.500	0.08
SR	0.032	0.798	0.862	0.935	521.899	1.917	0.054
ED	0.022	0.947	0.956	0.994	13.879	1.542	0.042
CLR-based business environment optimization	0.016	0.987	0.986	0.954	106.106	2.081	0.059

RMR, Residual Mean Root; GFI, Goodness of Fit Index; AGFI, Adjusted Goodness of Fit Index; CFI, Comparative Fit Index; CSV, Chi-Square Value; CSV/DF, Chi-Square Value/Degree of Freedom; RMSEA, Root Mean Square Error of Approximation.

Firstly, on ES Scale, RMSEA is 0.032, less than the fitness standard of 0.08. GFI is 0.798, close to the standard of 0.90. CFI is 0.935, higher than 0.90. Therefore, the model fits well. Secondly, on ED Scale, RMSEA is 0.022, far less than the fitness standard of 0.08. GFI is 0.947, greater than 0.90 of the standard. CFI is 0.956, much higher than the standard 0.90. Thus, the model fits well. Lastly, on the CLR-based business environment optimization Scale, RMSEA is 0.016, far less than the fitness standard of 0.08. GFI is 0.987, greater than 0.90 of the standard. CFI is 0.986, much higher than the standard 0.90. Hence, the model fits well. In conclusion, the three scales have good structural validity and are suitable for data analysis.

(5) Confirmatory Factor Analysis (CFA)

**Figure 7:**
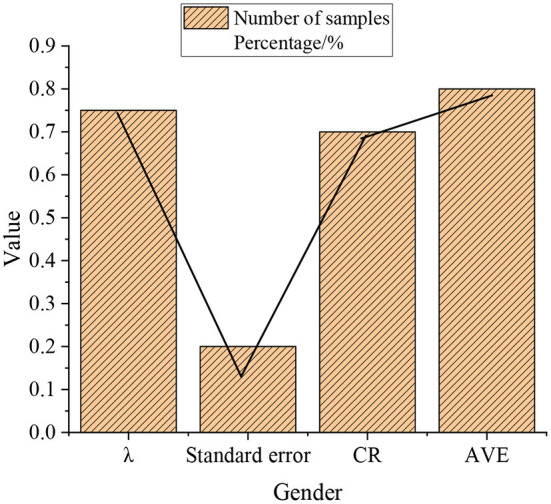
Results of CFA.

CFA is conducted on the three scales of CLR-based business environment optimization, ES, and ED. The standardized value λ is calculated respectively. The larger the λ is, the stronger the explanatory ability of the potential variables to the measured variables, and the better the index reliability is. The larger the standard error is, the smaller the measurement error is, indicating that the potential variables have a stronger ability to explain the measured variables. Composite Reliability (CR) represents the internal consistency reliability calculated by factor load. It uses the square of sums of factor load values. The stronger the correlation between items is, the stronger the explanatory ability of potential variables, the greater the square of the sum of factor load values, and the better the internal consistency is. Meanwhile, the Average Variance Extracted (AVE) represents the convergence validity calculated by the factor load. AVE value uses the sum of the squares of the factor load values to represent the comprehensive explanatory ability of the potential variables to all the measured variables. The larger the AVE is, the stronger the potential variables' ability to explain their corresponding items simultaneously. Conversely, the stronger the ability of the items to represent the nature of the potential variables (converging to one point) is, the better the convergent validity is. The calculation results are portrayed in Figure 7:

As manifested in Figure 7, the standardized value of the three scales of CLR-based business environment optimization, ES, and ED is 0.75>0.7. The standard error score is 0.2 < 0.5, CR score is 0.7>0.6, and AVE is 0.8>0.5. Therefore, the three scales designed have good reliability and small measurement error. Potential variables have a strong ability to explain the measured variables. The internal consistency is good, and the item convergence effect is good.

### ES survey results

(1) The relationship between ES and ED

**Figure 8:**
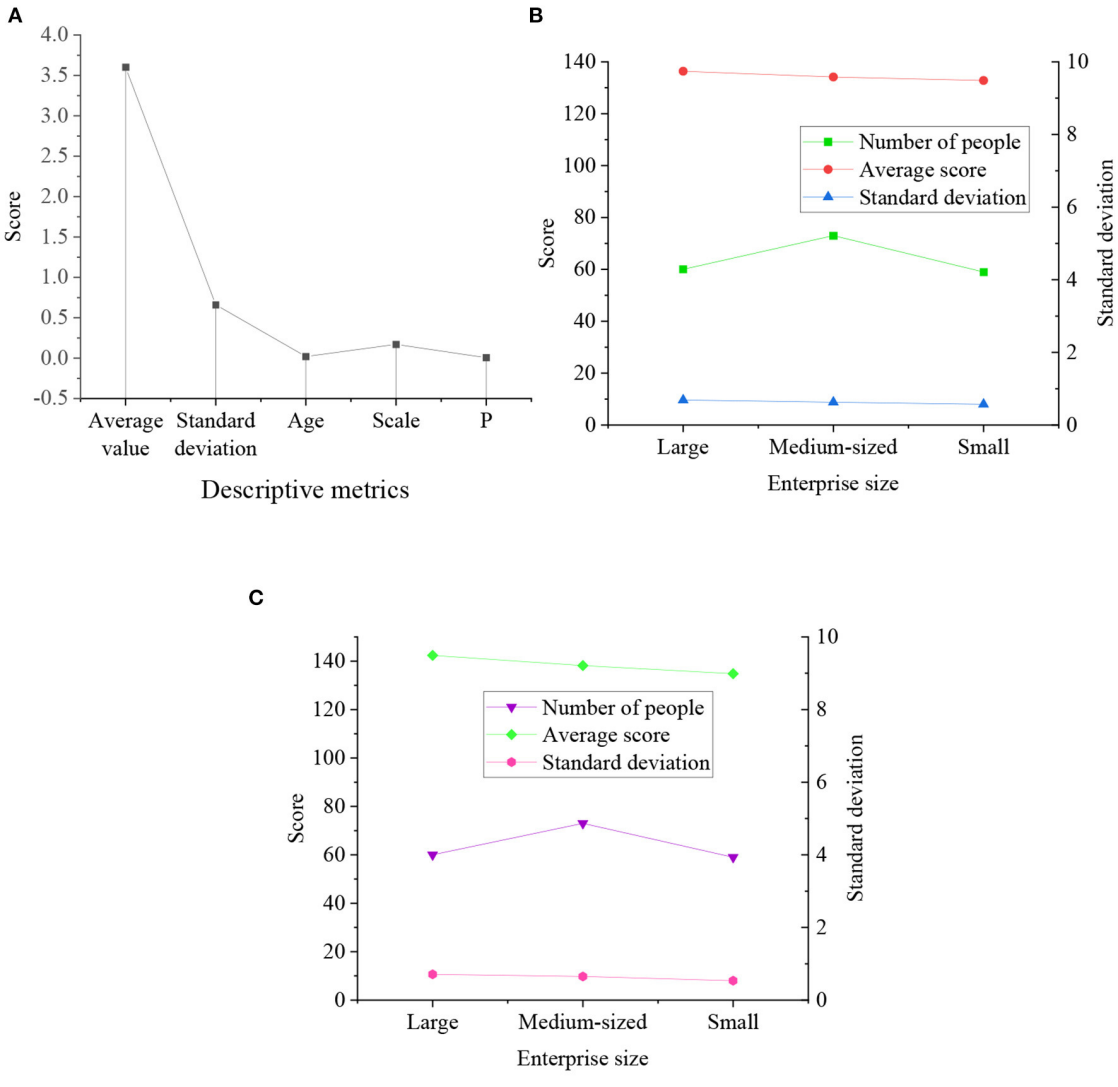
Results of the relationship between ES and ED **(A)** Descriptive statistical results; **(B)** Statistical results of ES of enterprises of different sizes; **(C)** Statistical results of the development of enterprises of different sizes.

The survey results of the relationship between ES and ED are explained in Figure 8:

According to Figure 8, the significance coefficient P of ES and ED is 0.005 < 0.01. Thus, ES and ED are significantly correlated. The average ES scores of entrepreneurs owning different-sized enterprises are 138.3687, 136.1434, and 132.7864, respectively. Apparently, the average ES score of large enterprises is the highest, and that of small enterprises is the lowest (only 132.7864). The average ED scores of entrepreneurs owning different-sized enterprises are 142.3243, 138.1432, and 134.6564, respectively. Obviously, the average ED score of large enterprises is the highest, and that of small enterprises is the lowest (only 134.6564). Therefore, the larger the enterprise-scale is, the higher the ES score is. The ES is conducive to the development of enterprise scale.

(2) Independent sample *t*-test results of ES

The independent sample *t*-test results of ES and ED are revealed in Table 2:

**Table 2:** Statistical table of independent sample *t*-test results of ES and ED.

**Factor**	***t*** **-test**						
	***t***	**df**	**Significance**	**Mean**	**Standard deviation**	**1–β**	***d***
/	3.320	421	0.001	2.63605	0.4401	0.968	0.30
ES	3,140	432	0.036	2.76453	0.45372	0.75	0.15
ED	3,432	463	0.043	2.65322	0.46821	0.83	0.25

According to the independent sample *t*-test results in Table 2, the significance between ES and ED is less than 0.01. Thus, the surveyed enterprises show a significant relationship between ES and ED. The *t*-test of different-sized enterprises' ES shows a significance = 0.036 < 0.05. The results indicate great ES differences among enterprises of different sizes. The statistical results of ED of different-sized enterprises show that the significance is 0.043 < 0.05. The result proves differences in ED of the enterprises with different sizes. The above two analyses show a significant positive correlation between ES and enterprise-scale development, and ES contributes to enterprise-scale development.

### Survey results of CLR-based business environment optimization

(1) The relationship between CLR-based business environment optimization, ES, and ED

**Figure 9:**
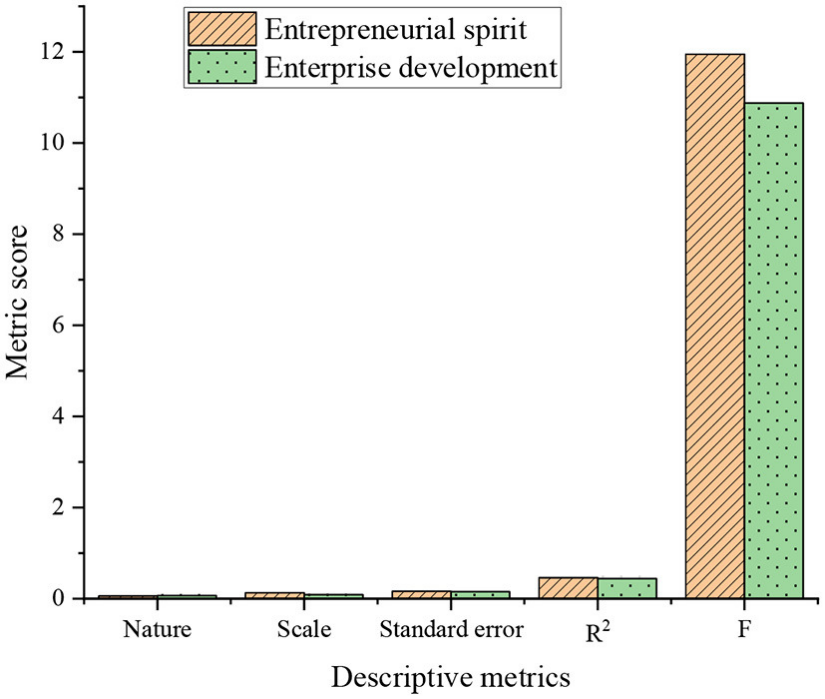
Descriptive statistical results of CLR-based business environment optimization, ES, and ED.

The descriptive statistical results of the business environment, ES, and ED are drawn in Figure 9:

As drawn in Figure 9, the influence coefficient between ES and CLR-based business environment optimization is 0.60, significantly positive. The direct influence coefficient of CLR-based business environment optimization and ED is 0.75, significantly positive. Therefore, o CLR-based business environment optimization has a significant positive impact on ES and ED.

(2) *t*-test results of CLR-based business environment optimization, ES, and ED

The *t*-test statistical results of CLR-based business environment optimization, ES, and ED are conveyed in Table 3:

**Table 3:** Statistical table of independent *t*-test results of CLR-based business environment optimization, ES, and ED.

**Factor**	***t*** **-test**							
	***t***	**df**	**Significance**	**Mean**	**Standard deviation**	**1–β**	***d***	**Sum of squares**
ES	3.125	418	0.042	3.2405	0.43401	0.753	0.15	614.027
ED	3.430	423	0.048	3.62453	0.53372	0.843	0.32	642.032

As from Table 3, the statistical results of CLR-based business environment optimization and ES are 0.042 < 0.05, which is significant. Therefore, there is a significant correlation between the two. The statistical results of CLR-based business environment optimization and ED are 0.048 < 0.05, indicating a significant correlation between them.

### Moderation analysis and test

**Figure 10:**
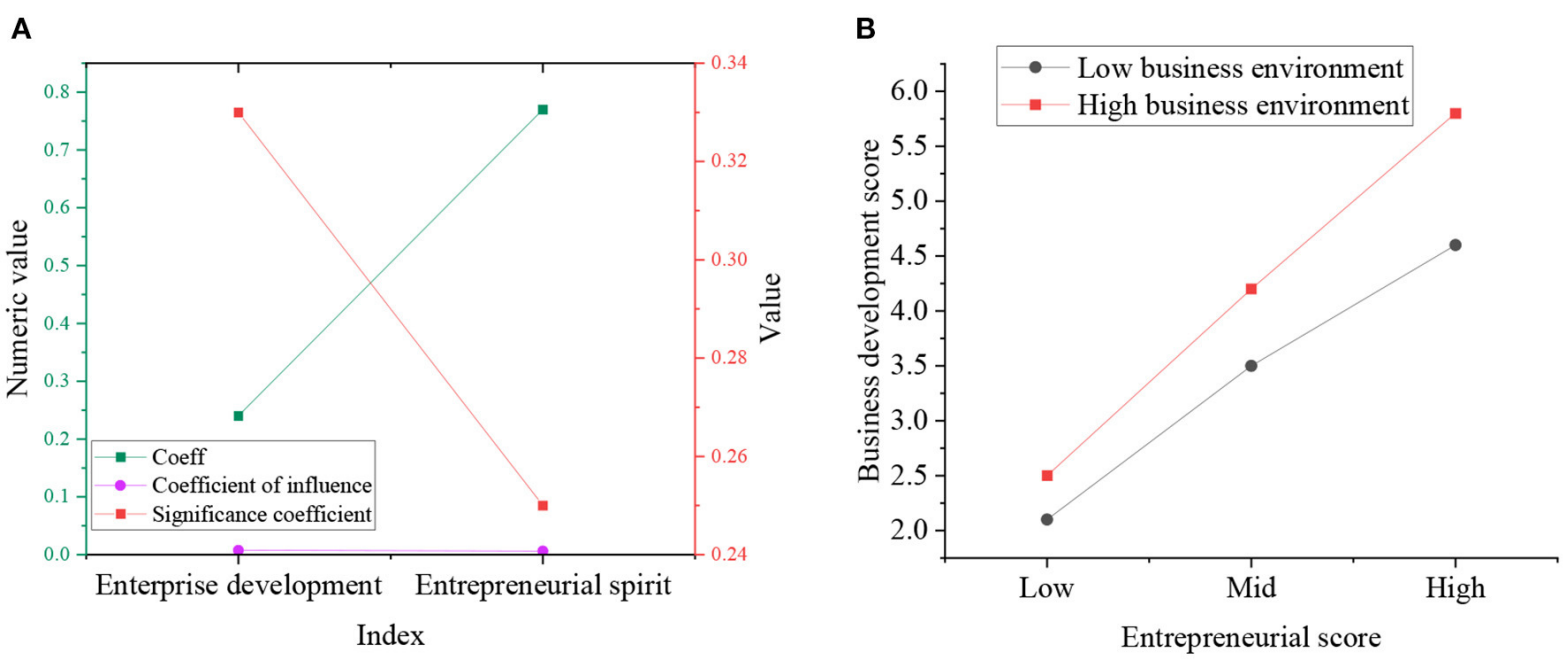
Bootstrap analysis result diagram **(A)** Index result diagram; **(B)** Relationship result diagram.

From the above analysis, CLR-based business environment optimization can affect ES and ED by playing a moderating role. This section uses the bootstrap method to test the moderating effect of CLR-based business environment optimization. First, it sets bootstrap to 40,00 and confidence to 96%. Then, it analyzes the impact of CLR-based business environment optimization on ES and ED at a high or low level. The analysis results are plotted in Figure 10:

In Figure 10, under the low CLR-based business environment optimization, the impact coefficient of ES on ED is small. By comparison, under the high CLR-based business environment optimization, the impact coefficient of ES on ED is large. Therefore, CLR-based business environment optimization positively impacts the relationship between ES and ED.

All in all, the hypothesis proposed is tenable. The following results are drawn. (1) CLR-based business environment optimization has a significant positive impact on ED. (2) CLR-based business environment optimization plays a positive role in the development of ES. CLR-based business environment optimization plays a moderating role between ES and ED. (3) CLR-based business environment optimization has a significant positive impact on the formation of ES.

## Conclusions

In order to study the impact of CLR-based business environment optimization on ES and ED, this work first expounds on the concepts and dimensions of the business environment and CLR. Secondly, it puts forward relevant hypotheses and a conceptual model. The QS method is selected to collect data. The statistical software is employed to analyze the reliability and validity of the CLR-based business environment optimization Scale, ES Scale, and ED Scale to construct the structural equation model. Specifically, this work discusses the impact of CLR-based business environment optimization on ES and ED through descriptive analysis. Consequently, the QS results corroborate that CLR-based business environment optimization has a significant positive impact on the ED and plays a positive role in the development of ES. Besides, CLR-based business environment optimization plays a moderating role between ES and ED and has a significant positive impact on the formation of ES. Lastly, the disadvantage of the research is that the selection range of research objects is narrow, which cannot involve different situations in different regions. Thus, the numerical result might somewhat have deviated. At the same time, the survey data cannot be targeted at all enterprises. Hence, sample data features temporal characteristics, and the data needs to be updated constantly to improve the model.

The experimental results show that (1) the average ED score of large enterprises is the highest. The average ED score of small enterprises is the lowest, only 134.6564. Therefore, the larger the enterprise scale is, the higher the ES score is, and the ES is conducive to developing the enterprise scale. (2) CLR-based business environment optimization has a significant positive impact on ES and ED. (3) This work analyzes the impact of the CLR-based business environment optimization on ES and ED in a high- or low-level business environment. According to the results, in the low business environment, the impact coefficient of ES on ED is small. In contrast, in the high business environment, the impact coefficient of ES on ED is large, indicating that the business environment has a positive regulatory effect on the relationship between ES and ED.

The policy enlightenment of this work mainly has three points. First, optimizing the business environment can stimulate and protect ES, thus promoting the quality of economic growth. All regions should promote mass entrepreneurship and innovation and high-quality economic development by improving the convergence of economic policies and building a legal and market-oriented business environment. Second, the spillover effect of improving the business environment on the TFP of most prefecture-level cities is still at a high level. While implementing the CLR-based business environment optimization, all regions should implement dynamic and differentiated policies to optimize the spatial pattern of the business environment in Chinese cities. Third, high-speed rail, Internet +, and other high-tech applications provide an important hardware foundation for optimizing the business environment. It helps to enhance the positive role of ES in China's economic operation in the new era. There is a need to strengthen further the construction of new infrastructures such as 5G business, big data, Artificial Intelligence (AI), and other cutting-edge information technologies. The aim is to better the growth of the urban business environment and ES and form a scientific and technological strategic force to promote high-quality economic development in the new era. The inadequacy of this work lies in the narrow selection range of research objects, which cannot involve different situations in different regions, and some data may be biased. At the same time, the survey data cannot be targeted at all enterprises, and the follow-up research needs to update the data to improve the model constantly.

## Data availability statement

The raw data supporting the conclusions of this article will be made available by the authors, without undue reservation.

## Ethics statement

The studies involving human participants were reviewed and approved by East China Normal University Ethics Committee. The patients/participants provided their written informed consent to participate in this study. Written informed consent was obtained from the individual(s) for the publication of any potentially identifiable images or data included in this article.

## Author contributions

All authors listed have made a substantial, direct, and intellectual contribution to the work and approved it for publication.

## Conflict of interest

The authors declare that the research was conducted in the absence of any commercial or financial relationships that could be construed as a potential conflict of interest.

## Publisher's note

All claims expressed in this article are solely those of the authors and do not necessarily represent those of their affiliated organizations, or those of the publisher, the editors and the reviewers. Any product that may be evaluated in this article, or claim that may be made by its manufacturer, is not guaranteed or endorsed by the publisher.
